# Protein Kinase C**ε**, Which Is Linked to Ultraviolet Radiation-Induced Development of Squamous Cell Carcinomas, Stimulates Rapid Turnover of Adult Hair Follicle Stem Cells

**DOI:** 10.1155/2013/452425

**Published:** 2013-04-29

**Authors:** Ashok Singh, Anupama Singh, Jordan M. Sand, Erika Heninger, Bilal Bin Hafeez, Ajit K. Verma

**Affiliations:** ^1^Department of Human Oncology, Wisconsin Institutes for Medical Research, School of Medicine and Public Health, 1111 Highland Avenue, University of Wisconsin, Madison, WI 53705, USA; ^2^Molecular and Environmental Toxicology Center, Wisconsin Institutes for Medical Research, Paul P. Carbone Comprehensive Cancer Center, School of Medicine and Public Health, University of Wisconsin, Madison, WI 53705, USA; ^3^UWCCC Flow Cytometry Core Facility, School of Medicine and Public Health, University of Wisconsin, Madison, WI 53705, USA

## Abstract

To find clues about the mechanism by which kinase C epsilon (PKC**ε**) may impart susceptibility to ultraviolet radiation (UVR)-induced development of cutaneous squamous cell carcinomas (SCC), we compared PKC**ε** transgenic (TG) mice and their wild-type (WT) littermates for (1) the effects of UVR exposures on percent of putative hair follicle stem cells (HSCs) and (2) HSCs proliferation. The percent of double HSCs (CD34+ and **α**6-integrin or CD34+/CD49f+) in the isolated keratinocytes were determined by flow cytometric analysis. Both single and chronic UVR treatments (1.8 kJ/m^2^) resulted in an increase in the frequency of double positive HSCs in PKC**ε** TG mice as compared to their WT littermates. To determine the rate of proliferation of bulge region stem cells, a 5-bromo-2′-deoxyuridine labeling (BrdU) experiment was performed. In the WT mice, the percent of double positive HSCs retaining BrdU label was 28.4 ± 0.6% compared to 4.0 ± 0.06% for the TG mice, an approximately 7-fold decrease. A comparison of gene expression profiles of FACS sorted double positive HSCs showed increased expression of Pes1, Rad21, Tfdp1 and Cks1b genes in TG mice compared to WT mice. Also, PKC**ε** over expression in mice increased the clonogenicity of isolated keratinocytes, a property commonly ascribed to stem cells.

## 1. Introduction

The multistage model of mouse skin carcinogenesis is a useful system in which biochemical events unique to initiation, promotion, or progression steps of carcinogenesis can be studied and related to cancer formation. 12-O-Tetradecanoylphorbol-13-acetate (TPA), a component of croton oil, is a potent mouse skin tumor promoter [1, 2]. A major breakthrough in understanding the mechanism of TPA tumor promotion has been the identification of protein kinase C (PKC), as its major intracellular receptor [3]. PKC forms part of the signal transduction system involving the turnover of inositol phospholipids and is activated by DAG, which is produced as a consequence of this turnover [3]. PKC represents a family of phospholipid-dependent serine/threonine kinases [3–6]. PKC*ε* is among the six PKC isoforms (*α*, *δ*, *ε*, *η*, *μ*, and *ξ*) expressed in both mouse and human skin [7]. We have reported that epidermal PKC*ε* levels dictate the susceptibility of PKC*ε* transgenic (TG) mice to the development of squamous cell carcinomas (SCC) elicited either by repeated exposures to ultraviolet radiation (UVR) [8] or initiation with 7, 12-dimethylbenz[a]anthracene (DMBA) and tumor promotion with 12-O-tetradecanoylphorbol-13-acetate (TPA) [9]. Histologically, SCC in TG mice, like human SCC, is poorly differentiated and metastatic [10]. 

SCC developed in PKC*ε* transgenic mice is metastatic and originates from the hair follicle [10]. The papilloma-independent carcinomas which develop in PKC*ε* transgenic mice arise from the hair follicle and have increased metastatic potential [10]. The difference in metastatic potential and the different origin of malignancy when compared to WT provided support for the hypothesis that papilloma-independent carcinomas in PKC*ε* TG mice were pathologically distinct from WT mouse carcinomas. Although the papilloma-independent carcinomas appeared to originate from the hair follicle, it was possible that the origin of the tumor was not within the hair follicle. The hair follicle might have been the easiest pathway for invasion. However, this did not appear to be the case because we observed neoplastic cells arising only from the hair follicle and not the epidermis. By harvesting PKC*ε* TG and WT mice after 8 weeks of DMBA + TPA or DMBA + acetone treatments, we identified possible premalignant areas in PKC*ε* transgenic mice as early as 8 weeks after DMBA + TPA treatment. The premalignant lesions originated within the hair follicle [10].

The metastatic potential of a transformed keratinocyte appeared to inversely correlate with the differentiation potential of that keratinocyte in the limited number of tumors studied to date. This conclusion was based on the location of invasion and pathological categorization of PKC*ε* TG mouse carcinomas compared with WT mouse carcinomas. Bulge keratinocytes are located near the sebaceous gland within the hair follicle. Evidence suggests that these cells appear to be the stem or progenitor cells for both the hair follicle and epidermis and, therefore, would be in a less-differentiated state than other epidermal cells [10]. These properties may increase the metastatic potential of these cells. The carcinomas of PKC*ε* TG mice that led to metastases were also less differentiated than carcinomas from WT mice. Evidence suggests that malignant cells invading from the hair follicle were less differentiated and had a higher metastatic potential than cells that invaded from the epidermis. PKC*ε*, when activated either via direct binding to TPA or indirectly by UVR treatment, mediates two potential signals leading to inhibition of apoptosis [11, 12] and induction of cell proliferation. 

Epidermal stem cells in the mouse hair follicle are known to be the precursor cells for SCC in the mouse skin [13–17]. Evidence suggests that epithelial stem cells reside in the bulge region [18, 19]. Stem cells, unlike transit amplifying cells, are slowly cycling and thus seem probable target cells. Moreover, stem cells may retain those mutations and pass them on to their progeny [14]. Morris et al. [20] demonstrated that label retaining cells (LRCs) have another property characteristic of potential initiated cells: they could retain carcinogen-DNA adducts. The contribution of follicular and interfollicular stem cells to the induction of skin papillomas and carcinomas was also determined [20]. Both follicular and interfollicular stem cells contributed to the development of papillomas. However, only follicular stem cells were linked to the development of carcinomas.

As a prelude to determine the SCC lineage from HSCs in PKC*ε* TG mice, we compared the responses of PKC*ε* TG and their WT littermates to UVR treatment. We examined the effects on proliferation, turnover, and gene expression profile of HSCs. In this communication, we present for the first time that (1) UVR exposures increased the number of double positive HSCs in TG mice, (2) the percent of double positive HSCs retaining BrdU label in the WT mice was 7-fold more than the TG mice, indicating that the double positive cells in the TG mice cycle at a faster rate, (3) the keratinocytes from PKC*ε* TG mice have higher proliferating potential compared to their WT littermates, and (4) a comparison of gene expression profile of FACS-sorted HSCs showed an increase expression of Pes1, Rad21, Tfdp1, and Cks1b genes in TG mice compared to their WT littermates.

## 2. Materials and Methods

### 2.1. Chemicals and Antibodies

BrdU was purchased from Sigma Aldrich (St. Louis, MO, USA). BrdU antibody was purchased from Santa Cruz Biotechnologies (Santa Cruz, CA, USA). Antibodies used for FACS such as *α*6-integrin PE-conjugated, CD34 FITC-conjugated antibodies, APC BrdU labeling kit, and propidium iodide were purchased from BD Biosciences (San Jose, CA, USA). BrdU antibody conjugated with Alexa Fluor 647 was procured from Biosciences (Frederick, MD, USA). PCR gene array focused to cell cycle was purchased from SA Biosciences (Frederick, MD, USA). 

### 2.2. Keratinocyte Isolation and Flow Cytometric Analysis

Keratinocytes were harvested as described elsewhere [21]. In each experiment an equal size of skin is excised from the WT and TG mice. Viable cell counts were determined using 0.4% Trypan Blue. Keratinocytes were incubated for 1 hr in the dark at 4°C with PE-conjugated Rat Anti-Human *α*6-integrin antibody at 10 *μ*L per 10^6^ cells and FITC-conjugated rat antimouse CD34 antibody at 2 *μ*g per 10^6^ cells (PE-*α*6-integrin and FITC-CD34 antibodies; BD Biosciences). Keratinocyte preparations were sorted based on *α*6-integrin+ and CD34 status using a FACS Aria cell sorter (BD Biosciences). Cells were stained with PE-conjugated anti-*α*6-integrin and FITC-conjugated anti-CD34 antibodies for flow cytometry. A 488 nm laser was used to detect FITC with a 530/30 filter and a 532 nm laser for PE with a 575/25 filter. The nozzle size was 130 nm and the pressure used was 14 p.s.i. The live cell population gate was estimated using forward and side scatter positioning and confirmed with 7AAD staining. 

### 2.3. Keratinocyte Colony Forming Assay

Keratinocytes were harvested from the dorsal skin of 7-8 weeks old PKC*ε* overexpressing mice (TG224 and TG215) and their WT littermates. The skin hairs were clipped and the skin pieces trypsinized for 2 hrs at 32°C. Epidermis was scraped in keratinocyte medium (SMEM) to isolate keratinocyte cells. Three thousand cells per dish were seeded onto irradiated 3T3 cells in 60 mm dishes and cultured for 2 weeks in high calcium medium. For feeder layer, 3T3 cells were cultured in EMEM medium with 10% FBS and 1% Penicillin-streptomycin and irradiated in Cesium Gamma Irradiator at 5000 rad. Irradiated 3T3 cells seeded 10^6^ cells/dish to the 60 mm dishes a day before seeding keratinocytes. The clonal culture was grown in William's E media with 10% FBS and supplements. For counting and measurement of colonies, dishes were fixed with 10% formalin for overnight. After fixation, the cultures were stained with 0.5% rhodamine B for 30 min to visualize colonies. The dishes were rinsed in cold tap water and dried before counting. The colonies were counted and colony size measured using vernier caliper.

### 2.4. Mice and UVR Treatment

WT and PKC*ε* TG 224 and 215 mice lines (FVB background) described elsewhere [9, 10] were housed in groups of two to three in plastic bottom cages in light-, humidity-, and temperature-controlled rooms; food and water were available *ad libitum*. The animals were kept in a normal rhythm of 12 h light and 12 h dark periods. The UVR source was Kodacel-filtered FS-40 sun lamps (approximately 60% UVB and 40% UVA). UVR dose was measured using UVX-radiometer. Mice were used for experimentation starting at 5 to 6 weeks of age. For 24 hr, 48 hr, and 72 hr time points, the mice were treated for 10 min (2 kJ/m^2^) and skin was harvested for keratinocyte isolation after UV exposure. However, for multiple or chronic UVR exposures, mice were exposed to UVR (2 kJ/m^2^) three times weekly (Monday, Wednesday, and Friday) or a total of 8 times. 

### 2.5. Detection of BrdU-Labeled Cells in the Hair Follicle Using Flow Cytometric Analysis

To identify the label retaining cells (LRCs), newborn mice (3 days old) were injected subcutaneously with BrdU (50 mg/kg body weight) twice daily for 3 days. There were three mice per group. Mice were sacrificed 3 to 8 weeks after BrdU injection (3, 4, 5, 6, and 8 weeks). Keratinocytes from the epidermis were harvested as described elsewhere [21]. Freshly harvested keratinocytes were incubated with PE-conjugated anti-*α*6-integrin and FITC-conjugated anti-CD34 antibodies, fixed and stained using the APC BrdU Flow Kit, following the manufacturer's instructions (BD Biosciences, San Jose, CA, USA). To prepare BrdU positive control samples for FACS, 5-week-old female mice were injected BrdU (50 mg/kg body weight) intraperitoneally for two days (2 times each). After two days, the spleen and thymus were harvested for BrdU positive cells and used as a positive control.

For cell cycle analysis, freshly harvested keratinocytes were isolated from mouse dorsal skin and stained with PE-conjugated *α*6-integrin and FITC-conjugated anti-CD34 antibodies. After surface staining, the cells were fixed and then stained overnight with DAPI for cell cycle analysis. Flow cytometric analysis based on *α*6-integrin, CD34, and BrdU was performed on a LSRII benchtop flow cytometer (BD Biosciences). A multilength ultraviolet laser along with a 450/50 bandpass filter was used to detect DAPI. DAPI was used for DNA staining for live/dead determinations along with cell cycle. For the detection of APC-conjugated anti-BrdU antibody, a 640 nm laser and a 660/20 filter was used. 

### 2.6. Phenotyping and Estimation of the Frequency of CD34+/*α*6-Integrin+ Stem Cells

The phenotyping assays were acquired on a BD FACSCalibur (BD Biosciences), and BrdU assays were acquired on an LSR II (BD Biosciences) benchtop flow cytometer. Both instruments were calibrated daily by the University of Wisconsin Carbone Cancer Center Flow Cytometry Laboratory staff using the manufacturer's Cytometer Settings and Tracking calibration software. Data were analyzed using FlowJo software version 9.4.3 (Treestar, Ashland, OR, USA). Positive staining and gating strategy were determined by comparison to isotype controls. Dead cells were excluded using 7-aminoactinomycin D (7AAD) staining on FACS Calibur assays or Invitrogen Live/Dead Fixable Violet (FLVD) staining for BrdU assays acquired on the BD LSR II. Data demonstrate frequency of cells in a parent population of live intact cells for *α*6-integrin and CD34 expression and of *α*6-integrin+/CD34+/live intact cells for BrdU incorporation.

The frequency of CD34+/*α*6-integrin+ stem cells represents the percent of CD34+/*α*6-integrin+/7AAD- cells (“cells” determined by FSC/SSC morphologic gate) in the total 7AAD population. The absolute number of CD34+/*α*6-integrin+ cells in individual samples was calculated by multiplying frequency of CD34+/*α*6-integrin+ stem cells by the total number of Trypan-Blue excluding cells in the single cell keratinocyte preparation. The data represent absolute number of CD34+/*α*6-integrin+ stem cells from the equal size of dorsal skin from WT and TG mice used in the study.

Similarly, for calculation for the absolute count of CD34+/*α*6-integrin+/BrdU+ cells, the total frequency of CD34+/*α*6-integrin+/BrdU+/FLDV-cells in total FLVD- was multiplied by the total counts of Trypan-Blue excluding cells in the single cell keratinocyte preparation.

### 2.7. Immunofluorescence Analysis

To identify the LRCs, newborn mice (3 days old) were injected subcutaneously with BrdU (50 mg/kg body weight) twice daily for 3 days. Mice were sacrificed at 3, 4, 5, 6, and 8 weeks after BrdU injection. Mouse skin was then excised promptly after euthanasia and immediately placed in 10% neutral-buffered formalin for fixation and then embedded in paraffin. Four to five *μ*m sections were cut for immunohistochemistry of BrdU and K15.

For immunofluorescence study extra paraffin was removed using three xylene gradient washes followed by alcohol gradient (95%, 90%, 70%, 50%, and 30%) for 10 min each. The slides were washed with Milli-Q water, and then 1XPBS. The antigen retrieval was done using antigen unmasking solution as per the protocol (Vector Laboratories). The blocking process was done in normal goat and normal horse serum for 1 hr at room temperature (RT). After blocking, primary antibodies (Keratin-15 monoclonal from Neomarkers, CA, dilution 1 : 30) and BrdU (Santa Cruz, dilution 1 : 50) were incubated to tissue section on the slides for overnight. Tissue sections were incubated with their secondary antibodies for 1 hr at RT such as Alexa-Fluor 488-Donkey antimouse IgG (H + L) and Alexa-Fluor 594-Donley antirat IgG (H + L) for k15 and BrdU from Invitrogen, respectively. After incubation with secondary antibody slides were washed three times with 1XPBS, mounted with DAPI, and observed under the fluorescent microscope (Vectra).

### 2.8. PCR Array and Real-Time PCR

TG mice and their WT littermates were exposed to UV once (2 kJ/m^2^), and 24 hrs after UV treatment mice were sacrificed and the dorsal skin removed for keratinocyte isolation. The cells were stained with fluorescent conjugated CD34 and *α*6-integrin antibodies and sorted. The cells were sorted into 5 mL tubes containing 0.5 mL of heat inactivated, chelated, fetal bovine serum. The collected cells were then spun down and placed into 300 mL of RNAprotect Cell Reagent (Qiagen; Valencia, CA, USA). RNA was isolated from double positive HSCs using SA Biosciences RT^2^ qPCR grade isolation Kit (SA Biosciences, Frederick, MD, USA). 250 ng RNA was used for first-strand cDNA synthesis with the SA Biosciences RT^2^ FirstStrand Kit. The resulting cDNA was used in the SA Biosciences Cell Cycle Gene Array according to the manufacturer's instructions.

The real-time expression primers of PKC*ε* in the study were selected from Origene website and further checked in NCBI primer blast for their primer-specific details such as proper target binding and amplification product (http://www.ncbi.nlm.nih.gov/tools/primer-blast/index.cgi?LINK_LOC=BlastHome). The RNA was isolated from FACS-sorted keratinocytes using Qiagen RNeasy mini kit. The samples were treated with DNAse to remove the DNA contamination using Qiagen RNase-free DNase set (Qiagen). For cDNA synthesis SuperScript First-Strand Synthesis Kit (Invitrogen) was used as per the manufacturer' protocol. 

Briefly a total of 50 *μ*L reaction mixture consisted of 25 *μ*L; 2X FastStart Universal SYBR Green master (ROX) mix, 30.0 *μ*M (1 *μ*L) forward and reverse primers, and PCR grade water, and 50–100 ng of cDNA (5–10 *μ*L) was used. Final volume of the reaction was adjusted with RNase-free water provided with the kit. The PCR was set up as per instrument protocol in MyiQ Biorad machine. A cycle to threshold (Ct) value was assigned automatically at the beginning of the logarithmic phase of real-time PCR. Finally, differences in Ct value of control (mouse Gapdh) and stem cell samples were used to determine the relative gene expression or fold changes of the PKC*ε*.

## 3. Results

### 3.1. Hair Follicle Stem Cells and Clonogenicity of Epidermal Keratinocytes Isolated from PKC*ε* Overexpressing Mice and Wild-Type Littermates

**Figure 1:**
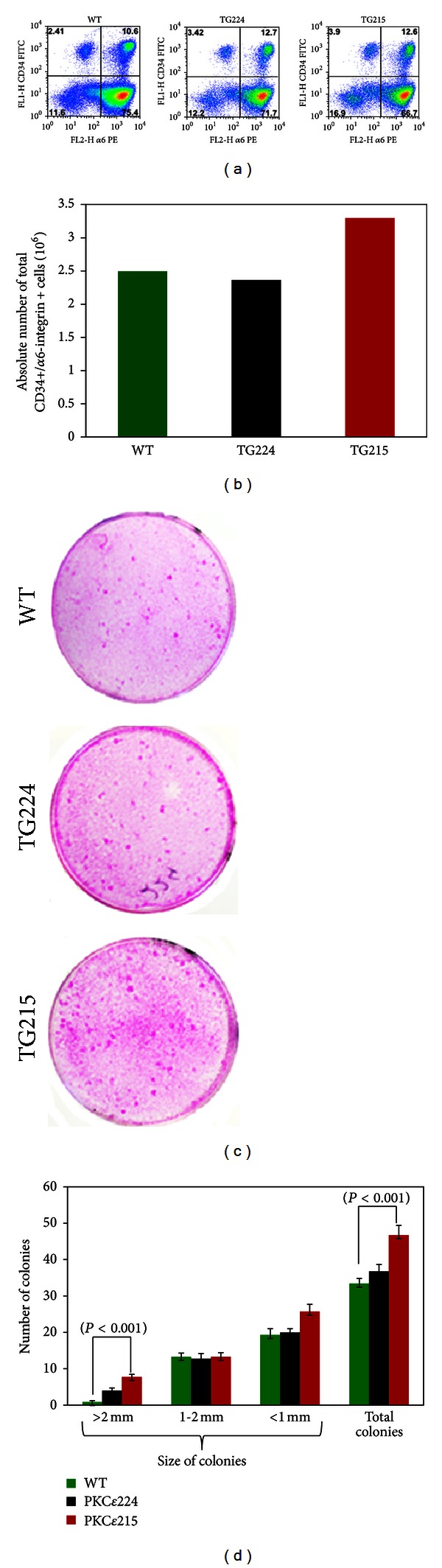
Hair follicle stem cells and clonogenicity of epidermal keratinocytes isolated from PKC*ε* overexpressing mice and wild-type littermates. (a) is showing the representative gating of epidermal stem cell population in a dot plot from untreated WT, TG224, and TG215 mice. In each dot plot, the upper right quadrant is representing the CD34+/*α*6-integrin+ (double positive HSCs) stem cell population. Each value in the histogram is an average of FACS analysis of triplicate samples from keratinocytes pooled from two mice. (b) represents the total frequency of CD34+/*α*6-integrin+ keratinocytes in untreated indicated mice. (c) and (d) Clonogenicity of epidermal keratinocytes. Briefly, the keratinocytes from 7-8 weeks old indicated that mice were harvested using SMEM harvesting medium. Irradiated 3T3 cells seeded at density 10^6^ cells/dish to the 60 mm dishes a day before seeding keratinocytes. For feeder layer, irradiated 3T3 cells were cultured in EMEM medium with 10% FBS and 1% penicillin-streptomycin. Equal numbers of keratinocyte cells (3000 cells/dish) were seeded for each type of mice and cultured with William's E media for 2 weeks. For counting and measurement of colonies, dishes were fixed with 10% formalin and stained with 0.5% rhodamine B. (c) Keratinocyte colonies. Shown are the representative dishes of adult keratinocyte colonies from PKC*ε* TG mice and their WT littermates. (d) Quantitation of colonies. The colonies were counted and colony size measured by using vernier caliper (Figure 1(c)). Each value is the mean ±SE of colonies from 4–7 dishes.

To determine the basal levels of double positive HSCs (CD34+/*α*6-integrin+) in untreated WT, TG224, and TG215 mice, freshly harvested keratinocytes were labeled with CD34 and *α*6-integrin antibodies and analyzed by flow cytometry for their total frequency. We observed higher frequency and absolute count of total HSCs in TG215 mice compared to WT and TG224 mice (Figures 1(a) and 1(b)). Furthermore, we determined the effects of PKC*ε* on the clonogenicity of keratinocytes. Clonogenicity is an intrinsic property of adult stem cells. In this experiment (Figures 1(c) and 1(d)), an equal number of isolated keratinocytes from WT, TG224, and TG215 mice were seeded onto the irradiated 3T3 (fibroblast) cells and left for two weeks. We observed increased colony formation in keratinocytes isolated from TG224 and TG215 mice compared with their WT littermates indicating more proliferative potential (Figures 1(c) and 1(d)). Notably, the colonies greater than 2 mm and total numbers of colonies were higher in TG215 mice compared to their WT littermates (Figure 1(d)). 

### 3.2. UVR Treatment Stimulates Putative Hair Follicle Stem Cell Proliferation

**Figure 2:**
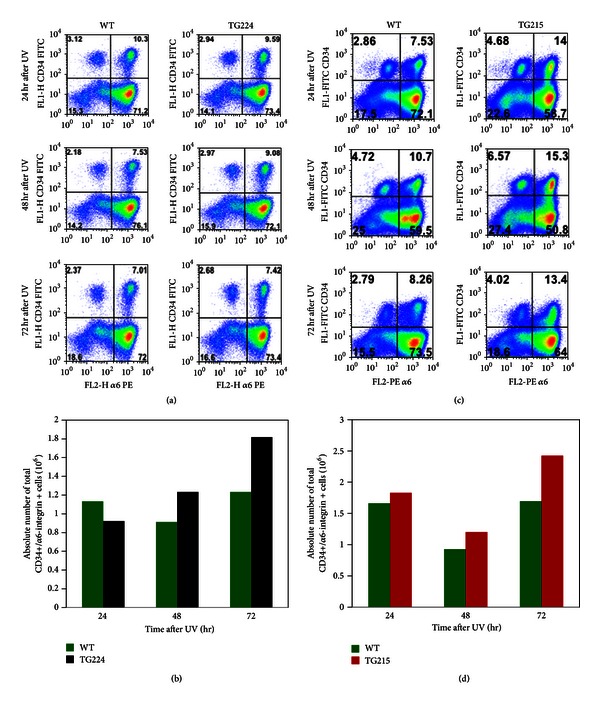
Effects of single UV exposure on live epidermal stem cell population determined by flow cytometric analysis. PKC*ε* overexpressing TG and their WT littermates were exposed once to UV (1.8 kJ/m^2^). At the indicated times after UV, mice were sacrificed and the dorsal skin removed for keratinocyte isolation as previously described [21]. (a) and (c) Percent distribution of FACS-sorted keratinocytes following UV exposure of the indicated mice at the indicated times after UV exposure. (b) and (d) Frequency of total double positive HSCs (CD34+/*α*6-integrin+) in TG224, TG215, and their WT littermates at the indicated times after single UV exposure.

**Figure 3:**
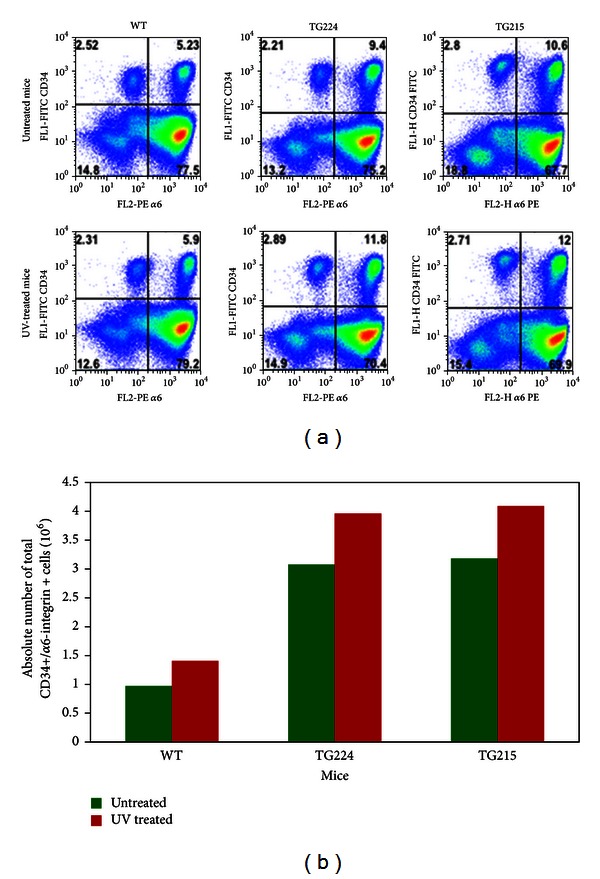
Effects of chronic UV exposures on live epidermal stem cell population determined by flow cytometric analysis. PKC*ε* overexpressing TG and their WT littermates were given total eight UV exposures (1.8 kJ/m^2^, Monday, Wednesday, and Friday). At 24 hr after the last UV exposure, mice were sacrificed and the dorsal skin removed for keratinocyte isolation. (a) Percent distribution of FACS-sorted keratinocytes following UV exposures of the indicated mice. (b) Frequency of total double positive HSCs (CD34+/*α*6-integrin+) keratinocytes in UV-treated mice.

 The cell surface markers CD34 and *α*6-integrin mark mouse hair follicle bulge cells, which have attributes of stem cells, including quiescence and multipotency. We determined the effects of UV treatment on total number of HSCs. In this experiment, PKC*ε* TG and WT mice were exposed to a single or chronic UV doses (1.8 kJ/m^2^, Monday, Wednesday, and Friday). At the indicated times after last UV exposure, mice were sacrificed and the number of putative HSCs was determined by flow cytometric analysis. The total frequency as well as absolute count of double positive HSCs were increased at 48 and 72 hr in TG224 after-UV exposure (Figures 2(a) and 2(b)) and 24, 48, and 72 hr in TG215 (Figures 2(c) and 2(d)) compared to their WT littermates. As shown in Figures 3(a) and 3(b), chronic UV exposures also showed an increase in total double positive HSCs in both TG224 and TG215 mice compared to their WT littermates. 

### 3.3. PKC*ε* TG Mice Have Increased Turnover of Putative HSCs

**Figure 4:**
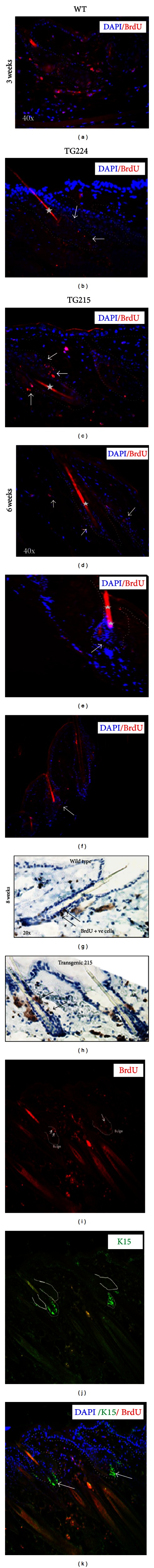
Detection of label retaining cells (LRC) by immunostaining using antibody to BrdU: to identify the LRCs, newborn mice (3 days old) were injected subcutaneously with BrdU (50 mg/kg body weight) twice daily for 3 days. Mice were sacrificed at 3, 6, and 8 weeks after BrdU injection. Shown are the representative photographs of BrdU-labeled cells from paraffin-fixed skin sections from WT and TG mice. The white arrow points to BrdU positive cells in the bulge region of hair follicle. In all the figures, the asterisk is the autofluorescence of the hair shaft. The incorporation and retention of BrdU are shown in the various parts of hair follicle at 3, 6, and 8 weeks in the indicated mice (a)–(h). (i)–(k) are showing the dual labeling of BrdU and k15 expressing cells in the bulge region of hair follicle.

 To determine the proliferation rates of bulge region stem cells in WT and TG mice, a 5-bromo-2′-deoxyuridine labeling (BrdU) experiment was performed. In this experiment, three-day-old neonatal mice were injected with 50 mg/kg of BrdU in PBS twice daily for three days. At 3, 4, 5, 6, and 8 weeks after BrdU injections, mice were sacrificed and the dorsal skin excised for keratinocyte isolation. Immunohistochemistry results revealed that, at 3-week time point, BrdU labeling was prominent in bulge region of hair follicle, interfollicular epidermis, and sebaceous glands of TG 224, 215, and their WT littermates (Figures 4(a)–4(c)). However, at later time points (6 and 8 weeks), BrdU labeling was decreased in TG mice compared to WT mice and localized to bulge region only (Figures 4(d)–4(h)). It is interesting to note that even after 3 weeks, some cells of interfollicular epidermis are able to retain the BrdU label (Figures 4(a)–4(c)). Moreover, dual immunofluorescence staining of BrdU and k15 indicates the colocalization of BrdU with k15 expressing cells in the stem cell-specific compartment, that is, bulge (Figures 4(i)–4(k)).

**Figure 5:**
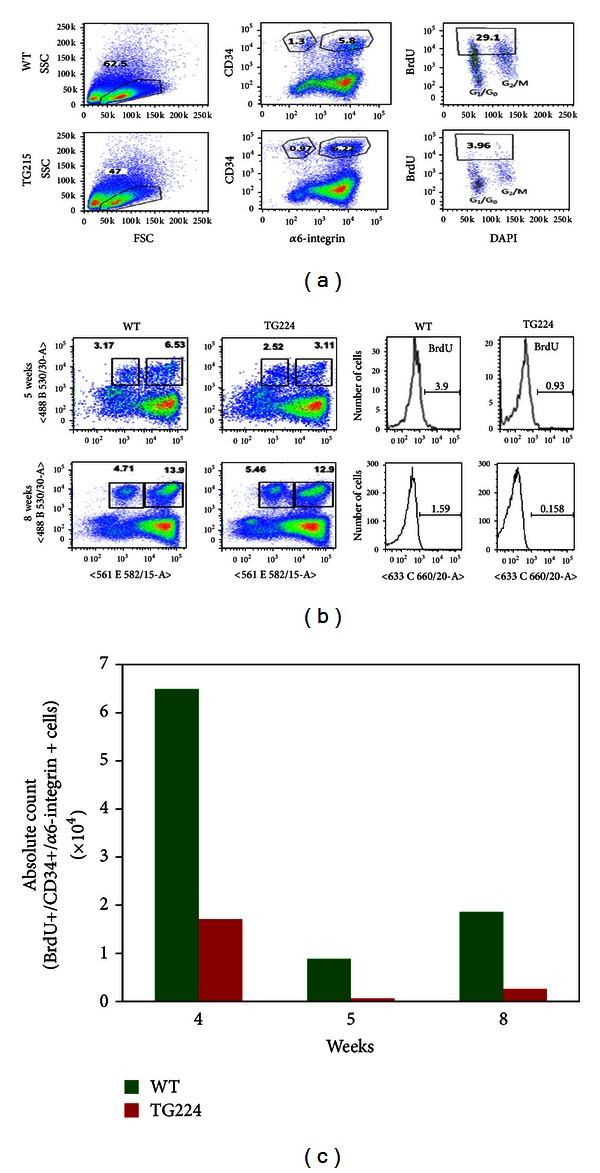
PKC*ε* overexpressing transgenic mice have increased turnover of HSCs as determined by BrdU retaining double positive HSCs (CD34+/*α*6-integrin+). To identify LRCs, newborn mice (3 days old) were injected subcutaneously with BrdU (50 mg/Kg body weight) twice daily for 3 days. Mice were then sacrificed at 8 weeks after the last BrdU injection. There were four mice per group. (a) Representative gating of cell populations for side scatter (SSC) versus forward scatter (FSC). (b) Representative gating of cell populations at 5 and 8 weeks in the indicated mice. (c) Frequency of total BrdU-labeled double positive (CD34+/*α*6-integrin+) keratinocytes in TG 224 and their WT littermates at 4, 5, and 8 weeks.

 We further evaluated the cell cycle pattern in sorted double positive HSCs in TG 215 and their WT littermates at 8 weeks after-BrdU injection. The percent of BrdU-labeled cells was different in WT and TG215 mice. In the WT mice, the percent of double positive cells maintaining BrdU label was 28.4 ± 0.6% compared to 4.0 ± 0.06% for the TG, an approximately 7-fold decrease (Figure 5(a)). 

 We further determined the turnover of HSCs in WT and TG 224 mice. In this experiment, we analyzed the BrdU retaining double positive HSCs in isolated keratinocytes from WT and TG mice. BrdU retaining cells were analyzed at 4, 5, and 8 weeks after-BrdU injections. The frequency of BrdU retaining double positive HSCs was at 4 weeks (WT = 0.246%, TG = 0.0807%), 5 weeks (WT = 0.0364%, TG = 0.00337%), and 8 weeks (WT = 0.167%, TG = 0.008%). There was also a decrease in total frequency as well as absolute count of BrdU retaining double positive HSCs in TG224 mice compared to WT littermates (Figures 5(b) and 5(c)). 

### 3.4. PKC*ε* mRNA Levels in FACS-Sorted Keratinocytes

**Figure 6:**
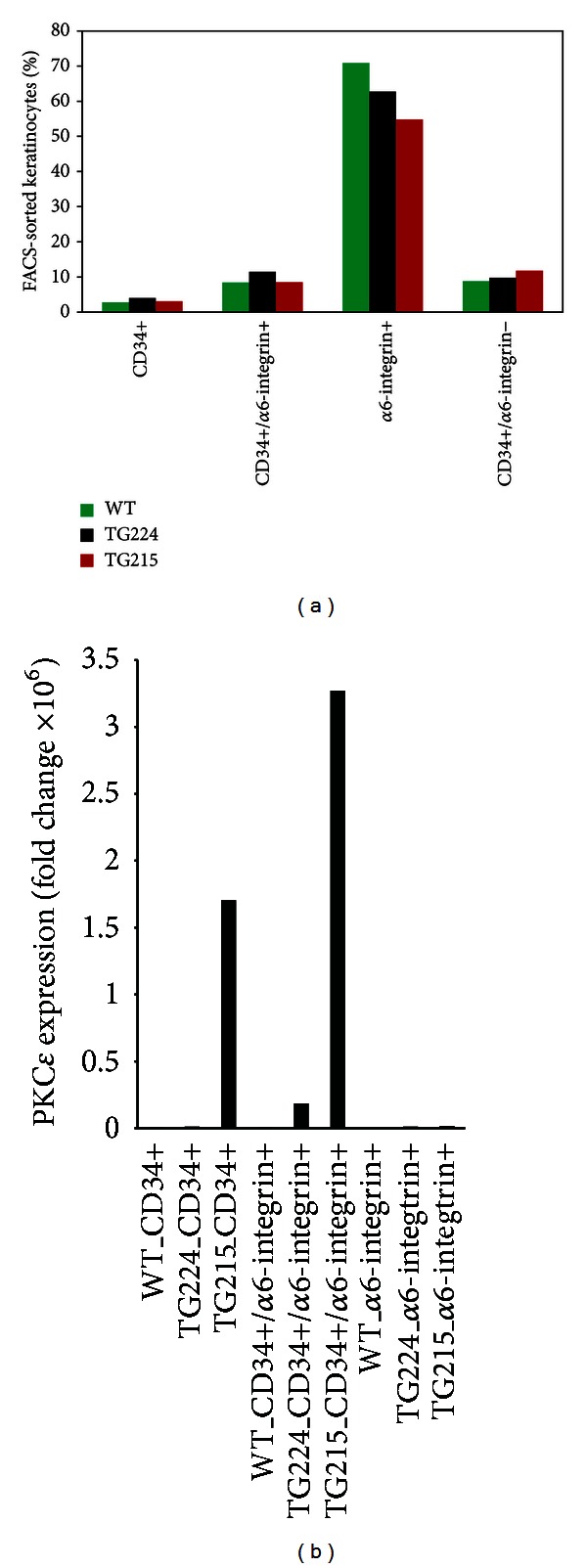
Distribution and expression of PKC*ε* in FACS-sorted keratinocytes from WT, TG224, and TG215 mice. (a) FACS-sorted keratinocytes. The keratinocytes were harvested from 5 weeks old wild-type and TG mice and incubated with the CD34 and *α*6-integrin florescent antibodies. After labeling, the cells were washed twice, filtered, and sorted for CD34+, *α*6-integrin+ cells, CD34+/*α*6-integrin+, and CD34−/*α*6-integrin-cells. (b) PKC*ε* expression. The RNA was isolated from the sorted cell, followed by cDNA preparation, and then real-time PCR using SYBR Green double-strand DNA binding dye. After real-time PCR Ct values were calculated and analyzed for expression. All the expression values shown in the figures are relative to their mouse Gapdh internal control.

 We first analyzed the percent distribution of CD34+, CD34+/*α*6-integrin+ (double positive HSCs), *α*6-integrin+, and CD34−/*α*6-integrin- (double negative) in TG224, TG215, and their WT littermates. The *α*6-integrin+ cells were 70.9%, 62.7%, and 54.8% in WT, TG224, and TG215 mice, respectively. The CD34+ cells were 2.8%, 4.0%, and 3.1% in WT, TG224, and TG215 mice, respectively (Figure 6(a)). We analyzed the PKC*ε* mRNA expression levels in FACS-sorted keratinocytes (Figure 6(b)). The higher expression of PKC*ε* was recorded in double positive HSCs of TG215 mice compared to WT and TG224 (Figure 6(b)). 

### 3.5. PKC*ε* Transgenic Mice Have Increased Expression of Genes Linked to Cell Transformation, Invasion, and Metastasis

A possibility explored that an increased turnover of HSCs in TG mice may be the result of changes in specific genes. In this experiment (Table 1), the effect of UV on cell cycle-related genes in double positive HSCs of TG and WT was determined using a focused cell cycle PCR array. Double positive HSCs of TG215 and their WT littermates were sorted out. A comparison of gene expression profiles of double positive HSCs is shown in Table 1. A 1.7- to 3.2-fold increase in the expression of Pes1, Rad21, Tfdp1, and Cks1b genes was observed in TG215 mice compared to their WT littermates. However, downregulation of Ccnf, Cdkn1a (p21), pkd-1, and Taf10 was observed in TG215 mice as compared to WT littermates.

## 4. Discussion 

Chronic exposure of Sun's UV radiation is linked to the development of human SCC, a metastatic nonmelanoma skin cancer [22]. We found using a novel PKC*ε* TG mouse model that the PKC*ε* levels in epidermis dictate the susceptibility of transgenic mice to the induction of SCC by UV [8]. PKC*ε* TG mice, when exposed to UV (2 kJ/m^2^ thrice weekly), elicited 3-fold increased SCC multiplicity and decreased tumor latency by 12 weeks. PKC*ε* overexpression in mice suppressed UV-induced sunburn (apoptotic) cell formation and enhanced both UV-induced hyperplasia and levels of specific cytokines (tumor necrosis factor *α* (TNF*α*), granulocyte colony-stimulating factor (G-CSF), granulocyte macrophage colony-stimulating factor, and interleukin six (IL-6)), implying inhibition of apoptosis and promotion of preneoplastic cell survival [8, 23]. Additionally, PKC*ε* may impart sensitivity to UVR carcinogenesis via its association with Stat3, a transcriptional factor that is constitutively activated in both mouse and human SCC [24]. We now present that PKC*ε*-mediated susceptibility to UV carcinogenesis may involve stimulation of putative HSCs proliferation possibly mediated by PKC*ɛ* and other specific genes linked to the cell cycle regulation. 

The epidermis undergoes a continual renewal throughout life, and the process is facilitated by various stem cell localized in both interfollicular epidermis and other specialized stem cell niches such as bulge. The skin stem cells present in different compartment of hair follicles respond differently to various signals mediated by their microenvironment [25]. Interestingly, multiple skin stem cell populations exist in the epidermis and play an important role during the process of controlled proliferation and differentiation (reviewed in [26]). The major stem cell population of hair follicle includes interfollicular label retaining cells (LRCs), double positive HSCs (CD34+/*α*6-integrin+), Mts24+ cells, Blimp1, Nestin, Lgr5+, and Lgr6+ cells (reviewed in [26]). However, the bulge region of hair follicle is considered as the major niche for keratinocyte stem cells [27, 28]. Particularly, the CD34+/*α*6-integrin+ cells are slow cycling and colocalize with LRCs and confined to bulge region of hair follicles. In terms of their colony forming ability (clonogenicity), CD34+ cells make larger colonies compared to CD34− cells [16, 29]. Keratinocytes isolated from PKC*ε* overexpressing TG have higher frequency of double positive HSCs (CD34+/*α*6-integrin+) and clonogenicity than their WT littermates (Figure 1). 

UV treatment resulted in a modest increase in total double positive HSCs in both TG224 and TG215 mice compared to their WT littermates (Figure 2). UV treatment in TG mice as compared to WT mice leads to constitutive activation of Stat3, increased Stat3-DNA binding [24], and increased expression of TNF*α* and G-CSF [8]. Results from genetic experiments indicate that both Stat3 and TNF*α* are linked to UV-induced development of SCC. It yet remains to be proven that PKC*ε* downstream components Stat3 and TNF*α* directly affect the proliferation of putative HSCs. 

We observed less BrdU retaining double positive HSCs cells in TG mice compared to WT (Figure 3). These results indicate rapid turnover of double positive HSCs cells in TG mice. Evidence indicates that at least two types of cell population exist: the slow cycling designated as stem cells and rapidly cycling cells as transit amplifying cells [30–32]. Rapid turnover of double positive HSCs cells in TG mice may be the effect of overexpression of PKC*ε* in TG mice and its associated cytokines such as TNF*α* and G-CSF [33]. Additionally, the level or percentage of BrdU retention in the stem cell populations is not consistently uniform. BrdU retention is influenced by mice age, time of labeling, and site of labeling [34]. This may be the possible explanation that the amount of BrdU retained varies in different repeat experiments (Figure 3). However, BrdU retaining double positive HSCs cells were consistently less in TG mice compared to WT. 

An analysis of focused cell cycle cDNA array revealed up- and downregulation of specific genes. The genes found to be overexpressed in double positive HSCs in TG215 were Pescadillo, Tfdp-1, Rad21, Nfatc1, Cks1b, AK1, and Itgb1. The gene Tfdp-1 is found to be overexpressed in SCC [35–38]. Interestingly, Nfatc1 gene is found to be overexpressed in many cancers, and its loss is linked with constant hair cycling and no quiescence [39]. Nfatc1 is also responsible for the balance between the quiescence and proliferation stage of skin stem cells [40]. Other overexpressed genes, Pescadillo, Rad21, Cks1b, Ak1, and Itgb1, are also linked with the process of carcinogenesis (Table 1).

## 5. Conclusion

In summary, we present for the first time an association of PKC*ε* with HSCs, the SCC precursors [20]. PKC*ε* overexpression in mice increased the clonogenicity of isolated keratinocytes, a property commonly ascribed to stem cells. Both single and chronic UV-treatments resulted in an increase in the frequency of double positive HSCs in PKC*ε* TG mice as compared to their WT littermates. In TG mice, HSCs cycle at a faster rate as compared to wild-type mice. A comparison of gene expression profiles of FACS-sorted double positive keratinocytes isolated from UV-treated WT and TG mice indicated increased expression of Pes1, Rad21, Tfdp1, and Cks1b genes in TG mice linked to cell transformation, invasion, and metastasis of cancer cells. 

It is believed that the skin stem cells are the major targets of carcinogen [15, 41]. However, the identification and the precise location of cancer initiating cells in cutaneous SCC is not clear. Furthermore, the role of nonstem cell cannot be overlooked during the process of carcinogenesis. It has been observed that the differentiated, nondividing epidermal cells with activated MAPK kinase 1 and inflammatory infiltrate can initiate benign tumor formation [42]. Interestingly, the differentiated keratinocytes can reenter into active cell cycling, dedifferentiating, and acquiring the stemness [43]. In future it will be interesting to study the link of double positive HSCs and other skin stem cell populations, along with other inflammatory signals in UV-induced Squamous cell carcinoma.

## Figures and Tables

**Table 1:** List of differentially expressed genes in CD34+/*α*6-integrin+ stem cells from PKC*ε* TG mice. Overexpression of PKC*ε* in the epidermis results in increased expression of genes linked to cell transformation, invasion, and metastasis.

Serial number	Gene name	Upregulated (↑) genes/fold change	Serial number	Gene name	Downregulated (↓) genes/fold change
(1)	Pescadillo	(3.2) ↑	(8)	Ccnf	(0.06) ↓
(2)	Tfdp-1 (Transcriptional factor)	(2.2) ↑	(9)	Cdkn1a (p21)	(0.5) ↓
(3)	Rad21	(1.8) ↑	(10)	Pkd-1	(0.5) ↓
(4)	Nfatc1	(1.7) ↑	(11)	Taf10 (TafII30)	(0.5) ↓
(5)	Cks1b	(1.6) ↑	(12)	Sfn	(0.6) ↓
(6)	Ak1	(1.3) ↑	(13)	Sumo1	(0.7) ↓
(7)	Itgb1	(1.2) ↑	(14)	RAN	(0.8) ↓

## Abbreviations

BrdU: 5-Bromo-2′-deoxyuridine
DAG: Diacylglycerol
DMBA: 7,12-Dimethylbenz[a]anthracene
FACS: Fluorescence assisted cell sorting
G-CSF: Granulocyte colony-stimulating factor
GM-CSF: Granulocyte macrophage colony-stimulating factor
HSCs: Hair follicle stem cells
LRCs: Label retaining cells
PKC*ε*: Protein Kinase C epsilon
SCC: Squamous cell carcinoma
TNF*α*: Tumor necrosis factor alpha
TPA: 12-O-Tetradecanoylphorbol-13-acetate
UVR: Ultraviolet radiation.
